# The impact of functional sagittal pelvic tilt on CT-based hip dysplasia diagnostics in adults: a cross-sectional 3D simulation study

**DOI:** 10.2340/17453674.2026.46974

**Published:** 2026-11-12

**Authors:** Milou F T HÜSKEN, Fianne LISSENDORP, Joëll MAGRÉ, Koen WILLEMSEN, Harrie WEINANS, Ralph SAKKERS, Jafar NASRABADI, Vahid ARBABI, Joris BEKKERS, Arno M ten HAM, Tom P C SCHLÖSSER, Bart C H van der WAL

**Affiliations:** 1 Department of Orthopedic Surgery, University Medical Center Utrecht, Utrecht; 2 Clinical Orthopedic Research Centre (CORC-mN), Diakonessenhuis, Utrecht/Zeist; 3 3D Lab, Division of surgical specialties, University Medical Center Utrecht, Utrecht; 4 Department Biomechanical Engineering, 3ME, TU Delft, Delft; 5 Department of Orthopedics, Sint Maartenskliniek, Nijmegen; 6 Department of Orthopedic Surgery, Leiden University Medical Center, Leiden, The Netherlands

## Abstract

**Background and purpose:**

Accurate acetabular coverage assessment is essential for diagnosing adult hip dysplasia. Computed tomography (CT) best evaluates 3-dimensional acetabular morphology but is typically acquired supine, while symptoms arise during weightbearing. This study evaluates how sagittal pelvic tilt affects CT-based acetabular parameters in adults with hip dysplasia and whether pelvic tilt causes clinically relevant measurement changes.

**Methods:**

3D-models from supine CT of 29 adults with hip dysplasia (lateral center–edge angle (LCEA) ≤ 25°) yielded 39 affected hips. LCEA, inclination (AI), anterior/posterior/horizontal acetabular sector angles (AASA, PASA, HASA), acetabular version (AV), extrusion index (EI), and total and loadbearing coverage were measured. Pelvic tilt was simulated in 5° increments from 30° anterior to 30° posterior around the hip centers and parameters were remeasured in each position. Differences < 2.5° were considered clinically irrelevant.

**Results:**

Median supine LCEA was 21°, AI 50°, AASA 43°. Across −30° to +30° tilt, AASA/loadbearing coverage decreased, PASA/AV increased, LCEA/EI showed parabolic patterns, and HASA/total coverage remained stable. From supine to simulated upright, paired mean differences were: LCEA −1° (95% confidence interval [CI] −2 to 0), AASA −4° (CI −5 to −3), PASA +4° (CI 4 to 5), HASA 0° (CI −1 to 1), AV +4° (CI 3 to 4), AI +1° (CI −1 to 3), EI +2% (CI 0 to 4), total coverage 0% (CI 0 to 0), and loadbearing coverage −2% (CI –2 to –2).

**Conclusion:**

Functional sagittal pelvic tilt resulted in generally small changes in CT-based acetabular measurements in dysplastic hips, which may be clinically relevant in borderline cases, particularly for AASA, PASA, acetabular version, and EI, whereas LCEA and AI were not significantly affected.

Hip dysplasia in adults is defined by insufficient femoral head coverage due to a shallow or misoriented acetabulum, resulting in increased joint stress, rim overload, and cartilage loss, and may be associated with early osteoarthritis [1-3]. Early diagnosis improves outcomes by enabling hip-preserving interventions before substantial degeneration occurs The severity of dysplasia is quantified by assessing femoral head coverage, rim deficiency, and acetabular depth using conventional or advanced 3-dimensional (3D) imaging.

Anteroposterior radiographs remain the standard diagnostic tool and are used to assess femoral head (sub)luxation and key parameters such as the lateral center–edge angle (LCEA). Dysplasia is indicated by an LCEA < 20°, and borderline dysplasia by 20–25° [4]. However, projection distortion causes radiographs to overestimate the LCEA by 2–3° compared with computed tomography (CT), reducing diagnostic accuracy, particularly in borderline cases [4,5]. As an alternative, CT enables detailed 3D assessment of hip morphology, including the anterior, posterior, and horizontal acetabular sector angles (AASA < 50°, PASA < 90°, HASA < 140°), acetabular version (AV < 19°), inclination (AI < 48°), extrusion index (EI > 25%), and load-bearing and total femoral head coverage (LC < 64%, TC < 44%), each with established thresholds (Table 1) [5-7].

**Table 1:** Baseline supine CT measurements, parameter definitions, and cutoff values

Parameter	Baseline supine CT value, median (IQR)	Description	Cutoff value
Lateral center–edge angle—LCEA (°)	21 (11–25)	Angle between a cranio-caudal line through the femoral head center and the lateral edge of the the acetabular rim in the coronal plane	> 25 is considered normal [4]
Anterior acetabular sector angle—AASA (°)	43 (30–48)	Angle between the line connecting the femoral head centers and the tangent to the anterior acetabular rim in the transverse plane	> 50 indicates sufficient anterior coverage [7]
Posterior acetabular sector angle—PASA (°)	87 (77–91)	Angle between the line connecting the femoral head centers and the tangent to the posterior acetabular rim in the transverse plane	> 90 indicates sufficient posterior coverage [7]
Horizontal acetabular sector angle—HASA (°)	129 (110–141)	Sum of AASA and PASA	> 140 indicates sufficient coverage [7]
Total femoral head coverage (%)	34 (30–38)	Percentage of the femoral head sphere surface covered by the acetabular rim	> 43.5 indicates adequate coverage [21]
Loadbearing femoral head coverage (%)	53 (46–55)	Percentage of the top hemisphere of the femoral femoral head sphere, defined by a horizontal plane passing through the femoral head center, covered by the acetabular rim	> 64 in normal hips [22]
Extrusion index—EI (%)	29 (25–40)	Percentage of uncovered femoral head compared compared with the total horizontal head diameter measured in the coronal plane	< 25 is considered normal [23]
Acetabular version—AV (°)	23 (20–29)	Angle between the line connecting the anterior and posterior acetabular margins and the perpendicular to a transverse reference line through the femoral head centers	Normal values around 19 ± 5 [24]
Acetabular inclination—AI (°)	50 (46–53)	Angle between the acetabular vector and the cranio-caudal line through the center of the femora head in the coronal plane	Normal values around 48 ± 6 [24]

However, CT is typically performed in the supine position, which does not reflect weightbearing, upright posture [6]. In patients with dysplasia, the pelvis generally tilts 5–6° posteriorly from the supine to upright position, rarely exceeding 10°, regardless of baseline orientation [8-11]. This sagittal pelvic tilt changes spinal alignment and directly affects parameters such as LCEA, AASA, and PASA, all of which are defined relative to the ground plane [4,6,10,11]. Consequently, positional changes may alter the parameters used in diagnosing hip dysplasia.

Studies in hips with normal angles confirmed that pelvic positioning impacts the accuracy of CT-based acetabular measurements [12,13]. However, dysplastic hips differ anatomically, and have not been evaluated. We therefore aimed to evaluate how sagittal pelvic tilt change CT-based acetabular parameters in adults with hip dysplasia, and whether pelvic orientations cause clinically relevant measurement changes.

## Methods

### Study design and imaging

Patients were retrospectively identified from CT scans in the St. Maartenskliniek electronic medical record (Nijmegen, the Netherlands) using the keyword “dysplasia” to filter radiology requests and reports since the year 2000. Adults with hip dysplasia or borderline hip dysplasia, defined as LCEA ≤ 25° on CT, were included. Exclusion criteria included incomplete pelvic imaging, prior pelvic osteotomies, or high dislocations (Hartofilakidis type C) that prevented defining the horizontal hip axis.

The study is reported according to STROBE guidelines.

### Image processing

Scans were acquired in the supine position for treatment planning. No standardized imaging protocol was applied.

Acetabular parameters were computed using custom software based on manually identified anatomical landmarks on pelvic CT scans by 2 observers (MH and FL) (Figure 1). These landmarks included the femoral head surface, acetabular rim, lunate surface, and sacral plate. The software generated digital reference points including the femoral head center, the 3, 9, and 12 o’clock rim positions, and the acetabular normal vector derived from the lunate surface. These reference points were used to calculate parameters including LCEA, AASA, PASA, HASA, load-bearing and total femoral head coverage, EI, AV, and AI. The exact definition of each parameter, baseline measurements, and cutoff values of these parameters is given in Table 1. Figure 2 illustrates the relevant anatomical landmarks.

**Figure 1:**
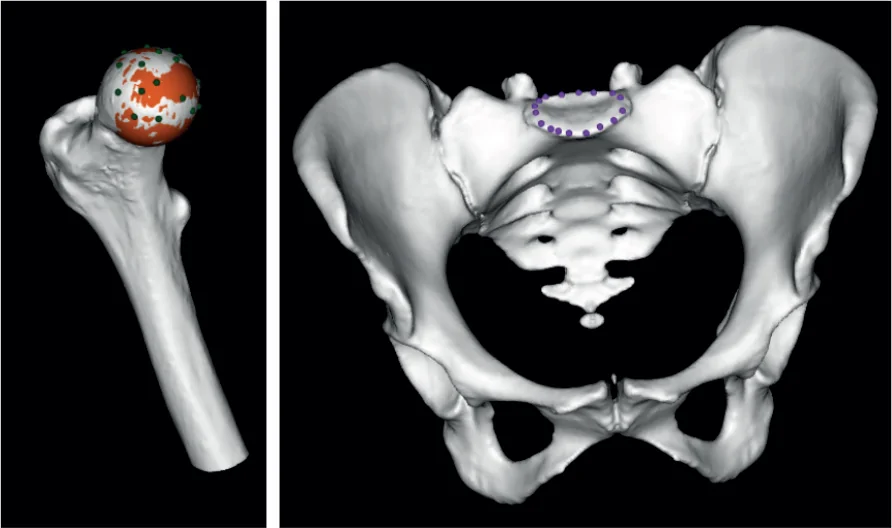
Example of manually identified anatomical landmarks. On the left identifying the surface of the femoral head to find a matching sphere and the center of rotation of the femoral head (used for several coverage parameters). On the right the rim of S1 to be able to find a matching plane to identify the angle of the S1 endplate (used for determining the pelvic tilt).

**Figure 2:**
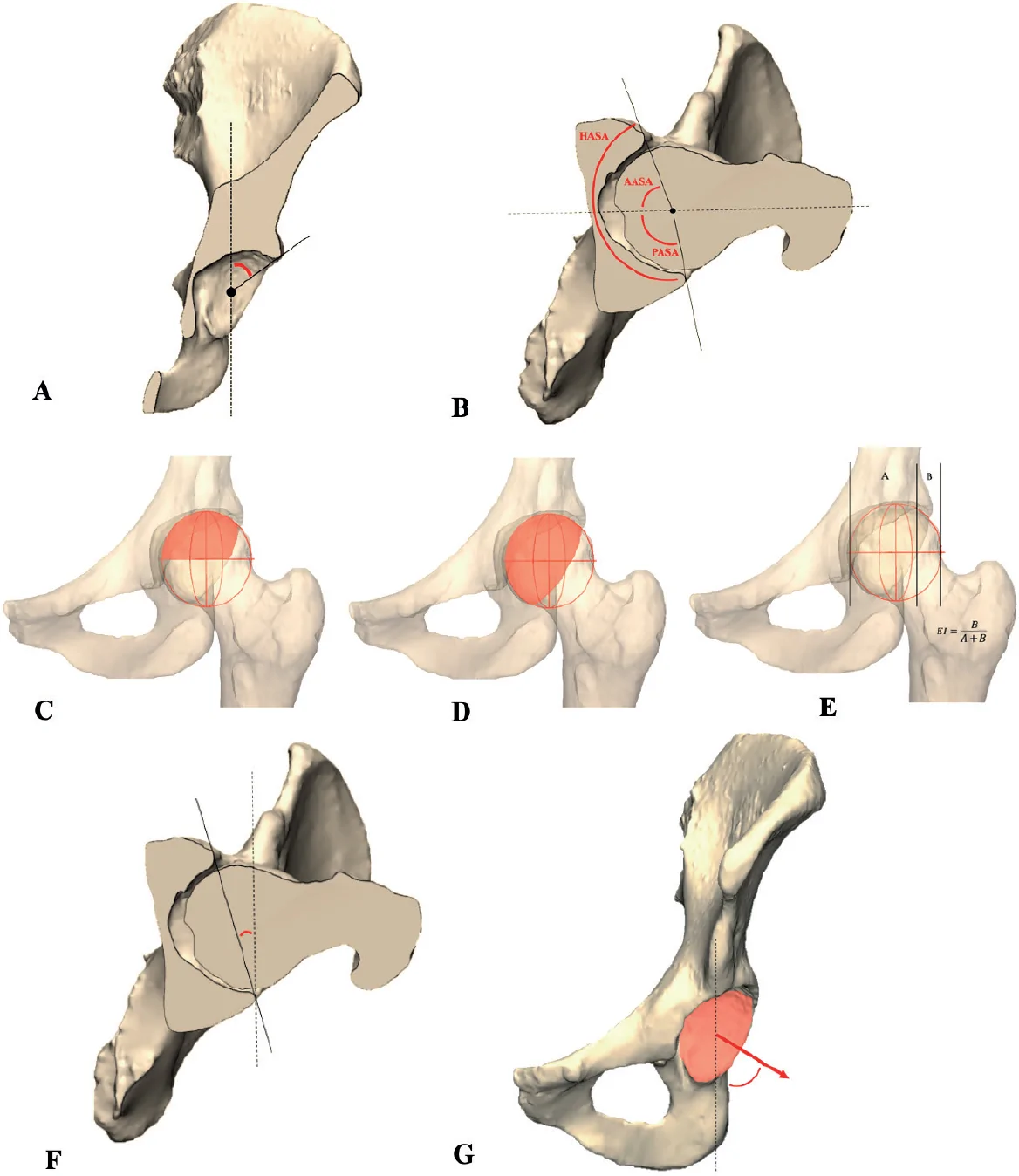
Visualization of the measurement techniques (see Table 1 for descriptives). (A) lateral center–edge angle (LCEA); (B) anterior, posterior, and horizontal acetabular sector angles (AASA, PASA, and HASA); (C) loadbearing femoral head coverage; (D) total femoral head coverage; (E) extrusion index; (F) acetabular version; and (G) acetabular inclination. For further descriptions, see Table 1.

3D segmentations were generated from preoperative CT scans using bone thresholding in Mimics (version 24.0, Materialise, Leuven, Belgium), with manual corrections by 2 orthopedic researchers (MH and JM). To account for pelvic asymmetry, a separate symmetrical pelvic model was created for each analyzed hip. The hemipelvis on the affected side was mirrored across the pelvic midline, defined by the sacral promontory and pubic symphysis. In patients with bilateral dysplasia, 2 separate models were generated, 1 for each hip, by mirroring the corresponding affected hemipelvis. The coronal plane was defined by a horizontal axis through the center of both femoral heads. Rotation of the pelvis in the transverse plane was corrected by aligning the anterior superior iliac spines. Sagittal orientation was preserved as captured in the original supine scan.

Pelvic tilt was defined as the angle between the vertical axis and a line connecting the midpoint of the S1 endplate to the midpoint between the 2 femoral head centers. To assess the effect of sagittal pelvic tilt on acetabular parameters, digital segmentations of the pelvis were virtually rotated in the sagittal plane around the horizontal hip axis, starting from the as-scanned supine position. The pelvis was tilted in 5° increments from 30° anterior to 30° posterior (Figure 3). The femurs remained in their original position to simulate pelvic movement during daily activities. The 10° posterior tilt simulation was used as a reference for the upright position. This value was based on prior studies reporting average posterior tilts of 5–6° during such transitions, with most patients demonstrating no more than 10° posterior tilt [8-11]. The 10° tilt therefore simulates a large but plausible change in pelvic tilt between the supine and upright positions in patients with hip dysplasia.

**Figure 3:**
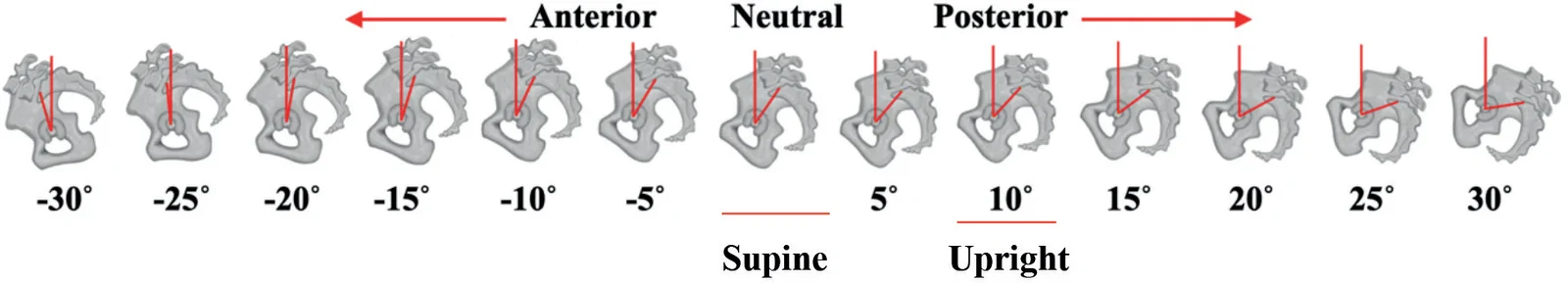
Subsequent changes in sagittal pelvic tilt of each model from the supine baseline position. For illustration purposes only, shown in an upright position instead of the supine position.

### Measurements and reliability

Intra- and interobserver reliability were assessed using intraclass correlation coefficients (ICCs) for input points and derived parameters. Intraobserver ICCs exceeded 0.95 for all parameters except acetabular inclination (AI), which showed an ICC of 0.54. Interobserver ICCs were all greater than 0.85.

### Statistics

No formal a priori sample-size calculation was performed. The number of patients was determined by the number of eligible CT scans available in the institutional database that met the inclusion criteria.

Statistical analyses were performed using SPSS (version 29.0; IBM Corp, Armonk, NY, USA). Data distributions were assessed for normality, and corresponding parametric or non-parametric tests applied. Each parameter was compared across pelvic tilt positions relative to the supine baseline position. To account for multiple testing, a Holm–Bonferroni correction was applied, adjusting the significance threshold for 12 comparisons per parameter. Relationships between acetabular parameters and sagittal pelvic tilt were visualized using locally estimated scatterplot smoothing (LOESS), a non-parametric method that displays data trends. Because each hip was measured in both the supine and simulated upright positions, the primary comparison was performed as a paired within-hip analysis, with each hip serving as its own control. For each hip, the paired difference was calculated as the value at 10° posterior pelvic tilt minus the value in the supine CT position. Paired mean differences are reported with corresponding 95% confidence intervals (CI). Differences smaller than 2.5° are considered unlikely to impact clinical decision-making in most patients [14]. For the supine and simulated upright positions, parameters were categorized according to published cutoff values (see Table 1), and changes in classification were recorded.

### Ethics, use of AI, funding, and disclosures

The study was exempt from ethical approval under the Dutch Medical Research Involving Human Subjects Act (WMO) and received a waiver of informed consent.

AI was used as a language model to edit and rewrite text; no AI was used to interpret or analyze data.

The research for this paper was financially supported by the PRosPERoS-II project, funded by the Interreg VA Flanders—the Netherlands program, CCI grant no. 2021TC16RFCB041.

MH was employed by Replasia B.V. from July 2024 until December 2024; VA was employed by Replasia B.V. during parts of this study and holds minority shares in Replasia BV. HW has received various research grants and contracts, holds minority shares in Replasia BV, Uplanner BV, and Amotio BV, and has patents pending in related areas. RS is listed as an inventor on several patents related to orthopedic implants and holds minority shares in UPlanner BV and Replasia BV. He also serves in leadership roles in the European Paediatric Orthopaedic Society and the Osteogenesis Imperfecta Federation Europe, among other boards. TS is a member of the EUROSPINE Executive Committee. BvdW holds patents related to orthopedic implants, has minority shares in Replasia BV, Amotio BV, and Uplanner BV, and is a member of advisory boards for these companies. The other authors declare no competing interests. Complete disclosure of interest forms according to ICMJE are available on the article page, doi: 10.2340/17453674.2026.46974

## Results

From 76 CT scans reviewed, 27 were excluded due to incomplete imaging, 11 due to prior surgery, and 9 due to high dislocations, leaving 29 scans (39 hips) for analysis (Figure 4). All variables showed non-normal distributions. There were no missing data for the variables included in the analysis.

**Figure 4:**
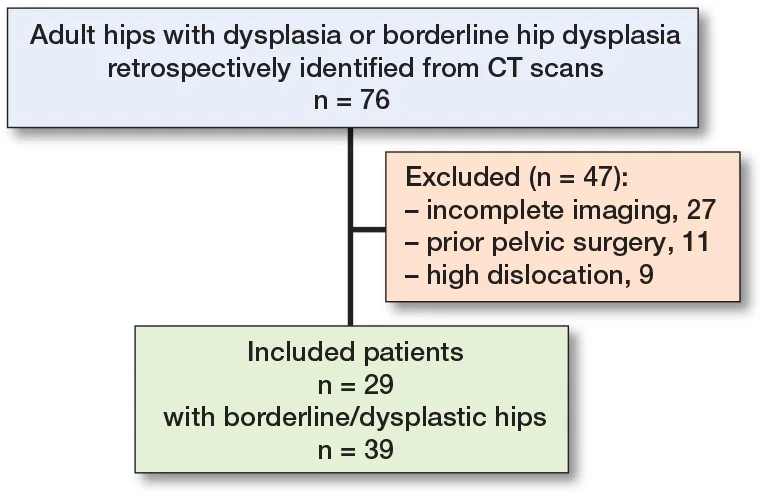
Flowchart of study inclusion. Of 76 CT scans reviewed, 47 were excluded, leaving 29 patients with 39 dysplastic hips for analysis.

### Changes across the full range of simulated pelvic tilt

In the supine position, the median LCEA was 21° (IQR 11°–25°), the median AI was 50° (IQR 47°–53°) the median AASA was 43° (IQR 30°–48°), and the median pelvic tilt in supine position was 9° (IQR 4°–17°). These values represent a range of dysplasia severity, including 16 (41%) cases of borderline dysplasia with a LCEA 20–25°. Detailed measurements are presented in Table 2.

**Table 2:**
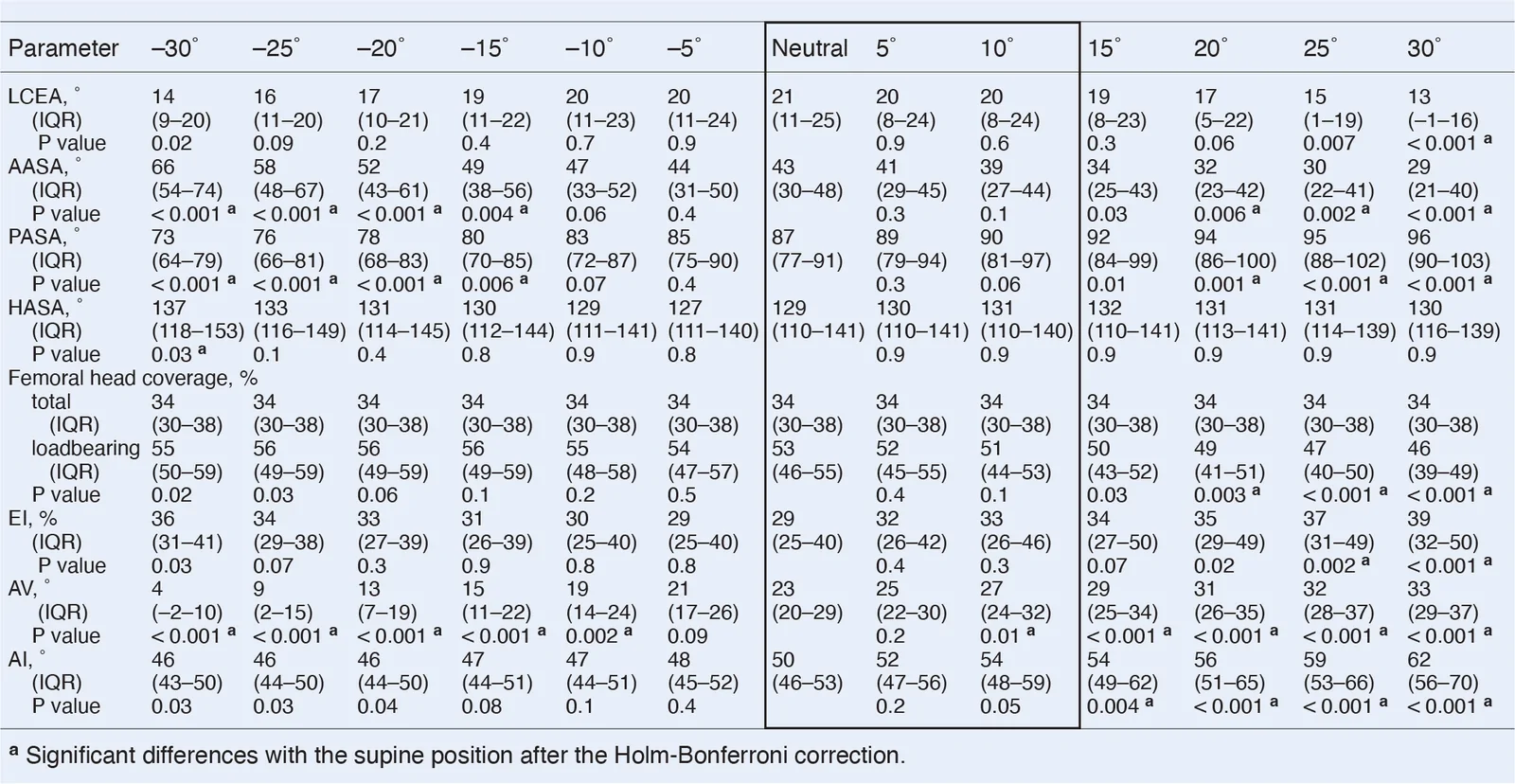
Comparison of pelvic parameters between different changes of pelvic tilt. Data is shown as median with interquartile range (IQR). The P value evaluates the difference from the supine position (Neutral). The outline indicates the pelvic tilt between supine (Neutral) and upright position (10˚)

Across the full sagittal pelvic tilt range (30° anterior to 30° posterior), most parameters showed a consistent increase or decrease (Table 2, Figure 5). The PASA increased continuously with posterior tilt, whereas the AASA declined, with the largest decrease between 30° anterior tilt and supine baseline, followed by a gradual decline. Loadbearing coverage also declined with increasing posterior tilt. Acetabular orientation parameters were more responsive to sagittal pelvic tilt than the coverage measures. AV increased notably with posterior tilt, from a median 23° at supine baseline to a median 33° at 30° posterior tilt.

**Figure 5:**
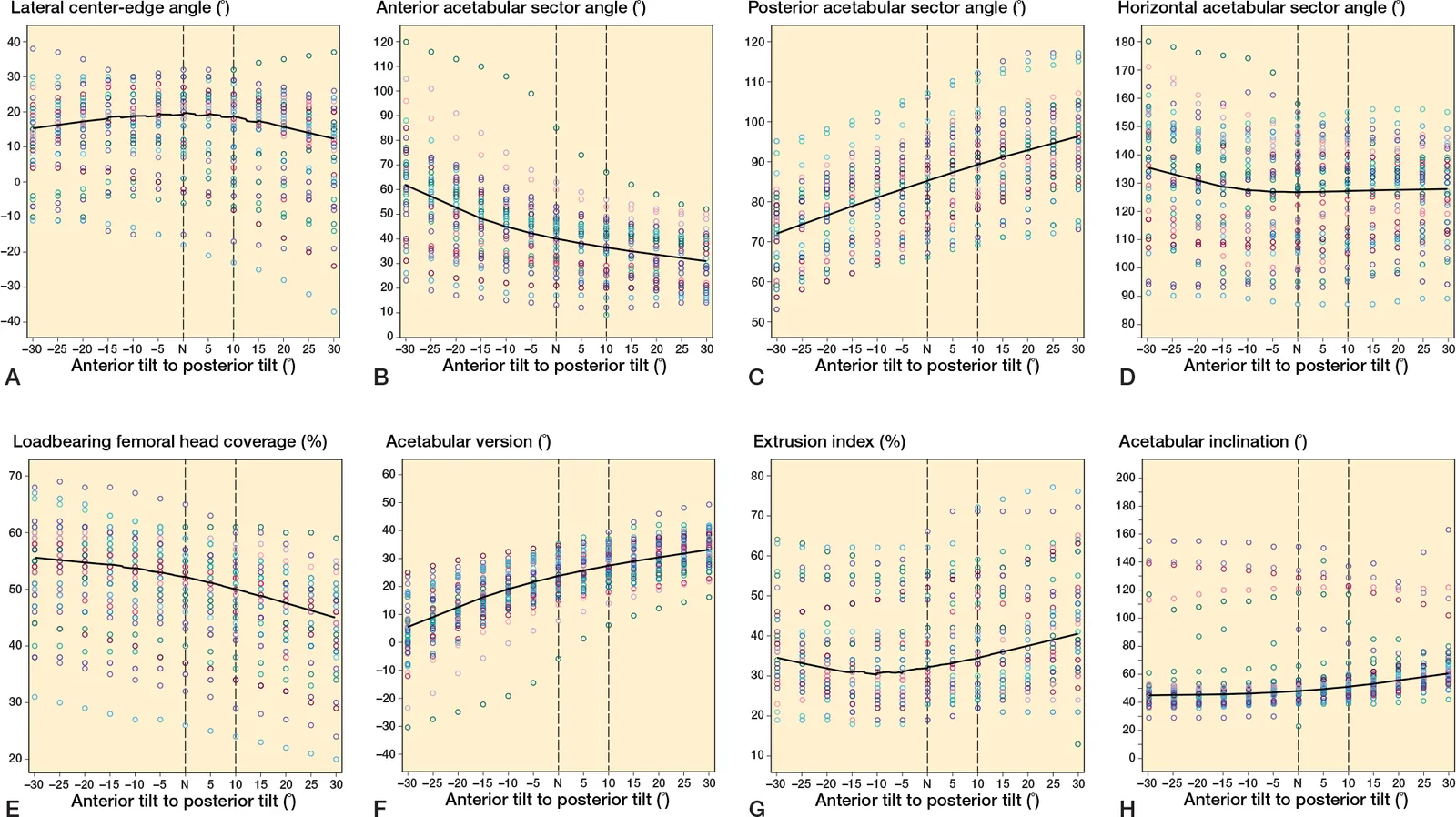
Scatter plots including a locally estimated scatterplot smoothing (LOESS) line across a range of sagittal pelvic tilt for the (A) LCEA, (B) AASA, (C) PASA, (D) HASA, (E) loadbearing femoral head coverage, (F) extrusion index, (G) acetabular version, and (H) acetabular inclination, with LOESS fit lines indicating the trend in the data. The vertical lines at the as scanned supine position (N) and the simulated upright position at 10° posterior tilt indicate the range within which the sagittal pelvic tilt changes.

The LCEA and EI, however, showed a parabolic-like pattern, characterized by a peak or trough near the supine position, as shown by the LOESS curves in Figure 5. The LCEA peaked around the supine position and decreased toward both anterior and posterior tilt, with a maximum reduction of 7° at 30° posterior pelvic tilt. Conversely, the EI showed its lowest value near 10° anterior tilt and increased again with further anterior or posterior tilting.

HASA and total femoral head coverage remained stable across the tilt range, with HASA 128°–132° and total femoral head coverage of about 34%.

From the supine position to the simulated upright orientation (10° posterior pelvic tilt), AASA, EI, AV, AI, and loadbearing coverage generally shifted toward values indicating more severe dysplasia, whereas PASA shifted toward values indicating sufficient posterior coverage. LCEA changed only slightly, while HASA and total femoral head coverage remained essentially stable (Table 3).

**Table 3:** Comparison of hip measurements in the supine position and simulated upright position with 10° posterior pelvic tilt. For the supine and simulated upright positions, values are reported as median and interquartile range (IQR). Paired differences are reported as mean difference with 95% confidence interval (CI) relative to supine. For each parameter and posture, the number and percentage of hips meeting reference “normal” cutoff values are provided

Parameter	Normal value	Supine position		Simulated upright position		Paired mean difference at 10˚ posterior tilt (CI)
		median (IQR)	normal values	median (IQR)	normal values	
LCEA, ˚	> 25	21 (11–25)	0 (0)	20 (8–24)	1 (3)	−1 (−2 to 0)
AASA, ˚	> 50	43 (30–48)	7 (18)	39 (27–44)	3 (8)	−4 (−5 to −3) **^a^**
PASA, ˚	> 90	87 (77–91)	8 (21)	90 (81–97)	18 (46)	4 (4 to 5) **^a^**
HASA, ˚	> 140	129 (110–141)	8 (21)	131 (110–140)	7 (18)	0 (−1 to 1)
Femoral head coverage, %						
total	> 44	34 (30–38)	0 (0)	34 (30–38)	0 (0)	0 (0 to 0)
loadbearing	> 64	53 (46–55)	0 (0)	51 (44–53)	0 (0)	−2 (−2 to −2)
EI, %	< 25	29 (25–40)	6 (15)	33 (26–46)	4 (10)	2 (0 to 4)
AV, ˚	< 19	23 (20–29)	9 (23)	27 (24–32)	4 (10)	4 (3 to 4) **^a^**
AI, ˚	< 48	50 (46–53)	12 (31)	54 (48–59)	7 (18)	1 (−1 to 3)

a Clinically relevant angular changes, defined as paired mean differences > 2.5°. Normal thresholds are defined according to values reported in the published literature (see Table 1 for references and abbreviations).

### Changes within functional changes in pelvic tilt

These positional changes resulted in the reclassification of some hips between the categories: hips with normal angles and hips with dysplastic angles. Several parameters were associated with shifts from a normal to dysplastic. The AI increased by 1° (CI –1 to 3), leading to 5 hips turning from normal to dysplastic values. AASA decreased 4° on average (CI –5 to –3), causing 4 hips to cross below the normal cutoff (> 50°). The EI increased by an average of 2% (CI 0 to 4), with 2 hips shifting to dysplastic. The AV increased by 4° (CI 3 to 4), reclassifying 6 hips to dysplastic. HASA changed by 0° (CI –1 to +1). 1 patient decreased in HASA below the normal cutoff (> 140°) and became dysplastic.

Conversely, PASA and LCEA were associated with shifts from a dysplastic to a normal classification in some hips. Although the LCEA decreased overall by 1° (CI –2 to 0), suggesting worsening dysplasia, the only true reclassification occurred in 1 patient showing an increase, reclassifying from dysplastic (≤ 25°) to normal (> 25°). PASA truly reclassified from dysplastic to normal in 10 hips, increasing by 4° (CI 4 to 5), causing those 10 hips to exceed the normal cutoff (> 90°). Total femoral head coverage remained stable (mean 0%) with no classification changes, and loadbearing coverage decreased by 2% (CI –2 to –2) without affecting classification (cutoff > 64%).

## Discussion

This 3D simulation shows that sagittal pelvic tilt affected CT-based acetabular parameters in adults with hip dysplasia, with the magnitude and direction of change varying between parameters. Between supine and simulated upright positions the LCEA and AI were not affected. For AASA, PASA, AV, and EI, changes were generally small, but clinically relevant variation could not be excluded, particularly in borderline cases. Across the full range of simulated pelvic tilt, most parameters showed non-linear changes. This is in line with the trigonometric, non-linear relations as described for the effect of pelvic tilt on a hemispherical cup in THA patients [15].

### Full range of pelvic tilt

Pelvic tilt primarily influenced acetabular orientation parameters rather than overall coverage measures. Total femoral head coverage and HASA remained stable throughout the tilt range, while loadbearing coverage decreased progressively with posterior tilt. The increase in PASA with corresponding decreases in LCEA and AASA shows that posterior tilt reduces anterior support, thereby decreasing loadbearing coverage. Together, these changes show that posterior tilt induces increased acetabular anteversion and inclination.

2 patients deviated from these general trends, showing peak LCEA values at extreme posterior tilt. Both had posterior wall deficiencies, dysplastic LCEA values (18° and 20°), elevated AASA, and low AV consistent with acetabular retroversion. In 1 case, retroversion aligned with native anterior pelvic tilt; in the other, retroversion appeared related to acetabular morphology. These cases indicate that isolated posterior wall defects alter parameter patterns differently from global dysplasia. This underscores the need to consider individual anatomical variation when evaluating hip dysplasia.

### Supine vs simulated upright

Our findings correspond to previously reported measurement error margins for acetabular parameters (±2° to 5°), attributed to patient positioning and variability in identifying anatomical landmarks [16,17]. Changes smaller than 2.5° are considered unlikely to affect clinical decisions [14].

In this study, LCEA, HASA, and loadbearing coverage shifted slightly toward more dysplastic values in the upright position, but all remained within measurement error (< 2.5°) and are unlikely to affect diagnosis or treatment. AI also changed with posterior tilt, but because intraobserver reliability for AI was low, these findings should be interpreted cautiously and should not be used as a primary basis for clinical conclusions. In contrast, the AASA, PASA, and AV showed larger positional changes. AASA decreased by 4° (CI −5 to −3), PASA increased by 4° (CI 4 to 5), and AV increased by 4° (CI 3 to 4), indicating that clinically relevant effects of simulated upright positioning cannot be excluded for these parameters. EI increased by 2 percentage points (CI 0 to 4), which may also be relevant when values are close to the diagnostic cutoff.

Given the median LCEA in this cohort was 21° (IQR 11–25°), reclassification is likely to be most relevant when baseline measurements are close to diagnostic cutoffs. Such shifts may influence clinical interpretation and treatment decisions when measurements are close to diagnostic cutoffs. These findings highlight that while most acetabular parameters are minimally influenced by patient positioning, parameters such as AASA, PASA, AV, and EI require particular attention in borderline cases. Careful consideration of measurement error and positional variation is important to avoid misclassification and inappropriate clinical decisions. The direct implications of sagittal pelvic tilt for surgical planning regarding periacetabular osteotomy seem to be of minor importance regarding LCEA and AI but changes in the retroversion may be significant. This should be investigated in future studies.

### Limitations

No formal sample-size or power calculation was performed. The results should be interpreted with caution, especially when drawing conclusions concerning smaller subgroups of patients. We did not focus on possible surgical implications within this study.

Although the scripted software showed high consistency across most parameters, challenges emerged for some measures. The parameter AI demonstrated lower intraobserver reliability (ICC = 0.54), because observers did not consistently record changes in the same direction. For example, in some cases the observer measured an increase while in another measured a decrease. Still, the mean absolute difference between observers was comparable to other parameters, indicating that the lack of agreement reflected directionality rather than a greater absolute error. Additionally, the distance between acetabular and femoral head centers showed lower correlation, reflecting observer variability in landmark approximation. Despite these issues, interobserver reliability remained high across varying dysplasia severity.

We deliberately selected the supine position on CT as the baseline for measurement, as it reflects a common imaging posture for CT and provides a stable, reproducible reference. This choice is supported by the substantial variability in spinopelvic tilt among patients with hip dysplasia [8,18]. Notably, regardless of baseline tilt, pelvic motion rarely exceeds 10° during postural changes [9,10], further supporting the supine position as a reliable reference for measurement. Because of the limited sample size, no formal stratified subgroup analysis based on baseline pelvic tilt was performed.

The inclusion criteria, based on LCEA and AASA, may have excluded atypical forms of dysplasia such as isolated posterior defects, thereby limiting generalizability. High dislocations (Hartofilakidis type C) were also excluded because the absence of a horizontal hip axis prevents accurate reference definition. In some patients, the contralateral dysplastic hip was still included. The affected hip was mirrored in all cases to assume symmetrical coronal alignment. Although mirroring reduces asymmetry, small discrepancies between original and mirrored hemipelves cannot be ruled out. With this technique no differences exceeding 3 mm or 2° are observed with 95% confidence [19], but smaller deviations may persist.

Simulating tilt as rotation around a single axis oversimplifies the true mechanics of dysplastic hips, where complex translations occur between the femoral head and acetabulum [20]. This introduces a methodological assumption, as postural changes involve more than tilt alone. True postural change may include axial loading, lumbar compensation, changes in femoral head position, pelvic rotation, and patient-specific spinopelvic mobility. Because the femurs were kept static and the pelvis was rotated around a fixed horizontal hip axis, the model should be interpreted as a controlled simulation rather than as a substitute for functional imaging. These limitations support the use of standing or dynamic (sitting/standing) imaging to better represent functional hip mechanics, and possibly in the context of surgical planning.

### Conclusion

Our 3D simulation shows that sagittal pelvic tilt affected CT-based acetabular parameters in adults with hip dysplasia, with the magnitude and direction of change varying between parameters. Between supine and simulated upright positions, changes were generally small, but clinically relevant variation could not be excluded for AASA, PASA, AV, and EI, particularly in borderline cases, whereas LCEA and AI appeared less sensitive to pelvic tilt. These findings suggest that pelvic orientation should be considered when interpreting borderline acetabular measurements, supporting the use of standardized upright imaging techniques or pelvic tilt correction of CT measurements.

## Notes

The study was conceptualized and the methodology was developed by MH, FL, JM, RS, HW, TS and BvdW. The software used for image analysis, 3D simulation and measurements was developed under the supervision of VA, who was responsible for its design, validation and testing; JN contributed to the software development. Data collection was conducted by AtH, MH, and JM. MH and FL performed the formal analysis of the data. The manuscript, including the creation of figures and charts, was written by MH and FL. The final manuscript was reviewed, edited, and approved by all authors. VA provided software development; supervision of the overall project was provided by RS, HW, TS, and BvdW.

The authors would like to thank Replasia BV for developing and providing the software essential to this study, along with their valuable technical support.

Handling co-editors: Paul Gerdhem and Robin Christensen

*Acta* thanks Geert Meermans and Katri Pernaa for help with peer review of this manuscript.
